# Exploring Adolescents’ Future Time Orientation: The Italian Validation of the Scale and Links to Sustainability

**DOI:** 10.3390/bs15030338

**Published:** 2025-03-10

**Authors:** Sara Santilli, Maria Cristina Ginevra, Vinicius Coscioni

**Affiliations:** 1 Department of Philosophy, Sociology, Education, and Applied Psychology, University of Padova, 35131 Padova, Italy; mariacristina.ginevra@unipd.it; 2 Department of Developmental Psychology, Utrecht University Heidelberglaan, 3584 CS Utrecht, The Netherlands; v.coscioni@uu.nl

**Keywords:** future time orientation, adolescents, sustainability, vocational guidance

## Abstract

Adolescents today face significant exposure to social inequalities and environmental crises, including the impacts of climate change, such as heatwaves, floods, and droughts. In addition, they encounter various forms of inequality, such as job insecurity, lack of affordable housing, and growing mental health challenges. Future perception is an essential variable in understanding how adolescents address these social and environmental challenges. Thus, this study adapted the Future Time Orientation Scale (FTOS) to the Italian context, assessing its validity and reliability for measuring psychological future orientation among Italian-speaking adolescents. Two studies were conducted: the first validated the FTOS through exploratory and confirmatory factor analyses, revealing two factors, “Impact” and “Distance”. The second study examined the relationship between future time orientation, future orientation in the professional field, and adolescents’ propensity to consider global challenges related to sustainable development. The results indicated that future orientation fully mediates the relationship between future time orientation and the tendency to consider global challenges in career decision-making. These findings underscore the importance of future orientation in shaping adolescents’ sustainable career choices and suggest that career interventions promoting future time orientation may enhance their engagement with global challenges.

## 1. Introduction

Adolescents today face increasing exposure to social inequalities and environmental crises, including the impacts of climate change, such as heatwaves, floods, and droughts (Thomaes et al., 2023). Simultaneously, they experience various forms of inequality, such as limited job opportunities, lack of affordable housing, cuts to public services, and rising mental health issues (Marmot et al., 2013). Although adolescents are not primarily responsible for these social and environmental challenges, they have the potential to play a key role in shaping educational and professional decisions that promote solutions to these pressing issues (Thomaes et al., 2023; United Nations Children’s Fund (UNICEF), 2021). In this regard, the COVID-19 pandemic, in addition to representing a phenomenon that strongly impacted adolescents’ psychological experiences and perceptions of the future (Commodari & La Rosa, 2020; Esposito et al., 2021), seems to have favored environmental awareness, sustainable consumption, and social responsibility in the population (Valenzuela-Fernández et al., 2022).

As a group, adolescents are increasingly concerned about global challenges and believe that addressing them promptly is essential. Many are motivated to contribute positively to society and are capable of making and supporting lifestyle changes with apparent ease (Thomaes et al., 2023). However, adolescents’ responses to these issues vary greatly. While some experience emotional distress, including anger, despair, and eco-anxiety related to climate change (Guichard, 2022; Kurth & Pihkala, 2022), others show lower rates of engagement in climate-relevant behaviors and do not consider social or environmental challenges when making decisions about their educational and professional futures.

Accordingly, future perception is an essential variable in understanding how adolescents address these social and environmental challenges (Wittmann & Sircova, 2018). The attention focused on said challenges and sustainability involves the personal and social commitment to take responsibility for the future results of current actions or, in other words, to have a future time orientation (Carmi & Arnon, 2014). This paper focuses precisely on future time orientation, defined as ‘the personal disposition to have the current psychological functioning impacted by the psychological future’ (Coscioni et al., 2024, p. 3). More specifically, this paper aims to adapt to the Italian context the Future Time Orientation Scale (FTOS), a recent questionnaire developed by Coscioni and colleagues (Coscioni et al., 2024; Gjesme, 1983) to assess the psychological future. It addresses some theoretical and statistical limitations of traditional instruments used in the literature to examine future time orientation. Moreover, using this questionnaire, this paper aims to explore the relationship between future time orientation and sustainability, focusing on the examination of adolescents’ inclination to consider global challenges and the idea of sustainable development connected to their future career choices through the mediational role of future orientation as the manifestation of future time orientation.

### 1.1. Future Time Orientation and the Future Time Orientation Scale

Future time orientation can be conceptualized as a personality trait, and specifically “a general capacity to anticipate, shed light on and structure the future, including a cognitive elaboration of plans and projects and reflecting the degree of concern, involvement and engagement in the future” (Gjesme, 1983, p. 452). Future time orientation can be differentiated by future orientation, that is, the ideas, thoughts, and feelings about the future in specific life domains. Future orientation is, therefore, a general manifestation of future time orientation regarding specific tasks (Coscioni et al., 2024; Topoğlu, 2022).

Both the constructs of future orientation and future time orientation are frequently utilized to delineate the aspects of the psychological future and are assessed by athematic and thematic methods, respectively. In particular, the future time orientation is generally assessed by athematic methods focusing on the general disposition toward the future without considering specific life domains (Coscioni et al., 2024; Seginer, 2009). On the contrary, future orientation is generally assessed by thematic approaches focusing on the psychological future within specific life domains such as professional careers (Coscioni et al., 2024; Seginer, 2009).

To evaluate future time orientation, an international team led by researchers from the Universidade de Coimbra, Portugal, and the Universidade Federal do Rio Grande do Sul, Brazil, along with scholars from China, Colombia, Finland, Israel, Italy, the Netherlands, Spain, and the USA, has recently designed the Future Time Orientation Scale (FTOS). The FTOS is a multidimensional measure that assesses the psychological future, allowing for a more extensive examination of the psychological future in contrast to other scales that focus on a single factor related to the future, such as the Zimbardo Time Perspective Inventory (Zimbardo & Boyd, 1999), the Consideration of Future Consequences Scale (Strathman et al., 1994), and the Time Orientation Scale (Holman & Silver, 1998). Moreover, in contrast to other multidimensional measures, such as the Future Time Orientation Measure (Gjesme, 1983) and the Future Time Perspective Scale (Shell & Husman, 2001), which exhibit some theoretical inconsistencies (e.g., in the Future Time Perspective Scale, the connectedness subscale includes items referring to long-term goals, which overlaps with the valence subscale; see for more information Coscioni et al., 2024) and statistical limitations (e.g., some items of the Future Time Orientation Measure showed reduced loadings to their subscales, and the total scale exhibited a reliability lower than 0.70; see for more information Coscioni et al., 2024), the FTOS shows good psychometric properties and has proven to be a valid and reliable tool. Across three studies with five independent samples, evidence for validity and reliability was determined through exploratory and confirmatory factor analyses, measurement invariance models, reliability coefficients, and relations to other measures (i.e., delay of gratification, concern). The FTOS was developed for people aged 12 to 65 years. It is a brief tool that consists of eight items loading onto two factors: the first factor is impact, which comprises five items that capture the inclination to be affected by the psychological future in current decisions and behaviors. The second factor is distance, involving three items that evaluate the perception of time in the future.

Despite the strengths of the FTOS, the authors underline the necessity of further studies to test the equivalence of the questionnaire across different cultures with more distinct historical backgrounds with respect to the Portuguese and Brazilian ones, as well as across different languages (Coscioni et al., 2024). Moreover, the authors highlighted the necessity to explore additional associations with distinct psychological constructs, especially for the distance factor. Therefore, the first study aimed to adapt the FTOS to the Italian context by examining its reliability, construct validity, and convergent and discriminant validity with the measures of future orientation, resilience, and career adaptability on a sample of Italian adolescents. The original element of the first study is the emphasis on cross-cultural adaptation and validation of the FTOS that responds to the gap in the current literature by offering empirical evidence on the scale’s applicability beyond its initial cultural environment. Adapting the FTOS to the Italian context can be useful to explore the construct of future time orientation in the Italian culture and measure differences across cultures (Epstein et al., 2015). Moreover, by adapting the FTOS to the Italian context, we may acquire in-depth knowledge regarding Italian adolescents’ perceptions of the psychological future and how they plan it, consequently facilitating the creation of targeted career interventions that encourage their career decision-making.

### 1.2. Future Time Orientation, Future Orientation, and Career Choices to Face Future Global Challenges

In the last decade, considerable attention has been focused on the concept of future orientation, particularly in relation to the promotion of sustainability (Wittmann & Sircova, 2018; Carmi & Arnon, 2014). Future orientation encompasses the ability to foresee and anticipate, make plans, and organize future options—skills that are fundamental for the individual and social assumption of responsibility in addressing global challenges (Carmi & Arnon, 2014; Vainio et al., 2020). Theoretically, this relationship is grounded in social and environmental global challenges, often framed as social dilemmas (Strathman et al., 1994). Social dilemmas arise when the short-term interests of individuals conflict with the long-term interests of society. Since current individual actions have collective future consequences, the time and social dimensions are inherently connected (Carmi & Arnon, 2014). Accordingly, several studies have found that higher levels of future orientation correlate with present behaviors and attitudes supporting sustainability (Wittmann & Sircova, 2018; Carmi & Arnon, 2014; Daysh et al., 2024).

In the career field, Thelken and de Jong (2020), for example, found the importance of future orientation and self-transcending values on the attitudes towards sustainable entrepreneurship when considering a sample of university students and, in turn, on the intention to decide to become a sustainable entrepreneur. According to the authors, the intention to make sustainable career choices (i.e., starting a sustainable company) is strictly connected to the ability to consider the future consequences of one’s future, that is to say, the willingness to consider the needs of future generations at the expense of their current benefits. This may be connected to the fact that higher levels of future orientation led to better preparation to soften the benefits of short-term advantages and, as a consequence, more conscientious decisions about the future (Muñoz, 2018). Even if such studies underlined the relevance of future orientation associated with the inclination to undertake sustainable careers, they have been carried out exclusively with young adults, usually university students, disregarding the relationship between the general disposition (future time orientation) and its manifestation (future orientation) and/or are focused on specific sustainable professional paths (i.e., sustainable entrepreneurship).

Nevertheless, the relationship between future time orientation, conceptualized as a general disposition toward the future (Coscioni et al., 2024); future orientation, understood as the ideas, thoughts, and feelings individuals have about their professional future (Di Maggio et al., 2016); and their connection to environmentally and socially sustainable career choices remains underexplored in adolescence. This distinction between the two constructs of future time orientation and future orientation warrants further investigation, particularly to understand how these constructs interact and influence sustainable career decision-making during this critical developmental stage.

Thus, the second study, considering an independent sample of adolescents, aims to examine the relationship between future time orientation, future orientation, and the propensity of adolescents to consider global challenges to attain sustainable development in their reflections about their future. More specifically, we expect that future orientation in the professional field mediates the relationship between future time orientation and the tendency of adolescents to consider global challenges to attain sustainable development in their reflections about their future careers.

## 2. Study 1

The first study aimed to adapt the FTOS to the Italian context, along with an analysis of its factor structure and internal consistency. This research aimed to detail the procedures for replicating the model obtained from exploratory and confirmatory factor analyses, as well as to gather evidence of convergent and discriminant validity with existing instruments to ensure its effectiveness in measuring psychological future orientation among Italian-speaking adolescents. We expected to confirm the original factorial structure found by Coscioni and colleagues (Coscioni et al., 2024).

Regarding the convergent and discriminant validity, we tested the correlations between FTOS and career adaptability (i.e., psychological skills useful to consider environmental eventualities to proactively adjust them to necessities and values in a career path; Career Adapt-Abilities Scale-Italian Form) (Savickas & Porfeli, 2012), resilience (i.e., the ability to cope with unexpected difficulties; Design My Future) (Di Maggio et al., 2016; Luthans et al., 2006), and future orientation (i.e., the ability to think about multiple possible future scenarios in professional future; Design My Future) (Di Maggio et al., 2016). We hypothesized weak correlations between the responses to FTOS and resilience and subscales of career control, curiosity, and confidence in career adaptability. We instead expected a moderate-to-strong correlation between the responses to FTOS and future orientation, and subscale career concern of the career adaptability, as these measures are more focused on the future.

### 2.1. Method

#### 2.1.1. Participants and Procedure

The sample size was determined a priori to ensure an adequate fit of the FTOS, starting with a translation of the full English version, which included a two-factor model with eight manifest variables. Using the root-mean-square error of approximation (RMSEA) as the measure of model fit, a minimum of 150 participants provides a 90% power level to test RMSEA ≤ 0.05 when RMSEA = 0.08, using a 0.05 significance level. This sample size was deemed adequate to ensure reliable parameter estimation in Exploratory Factor Analysis (EFA) and Confirmatory Factor Analysis (CFA) models. Specifically, for EFA, we ensured a minimum subject-to-variable ratio of 10:1, aligning with guidelines for robust factor analysis (Teo, 2009). For CFA, our power analysis was based on RMSEA estimation, with a target power of 0.90 to detect an RMSEA ≤ 0.05, assuming that our factor loadings would be moderate to strong (≥0.50). A sample of 150 was sufficient for a two-factor model with eight observed variables, ensuring stable parameter estimation.

Participants were 340 Italian high school students aged 15–19 years (Mage = 17.38; SD = 0.81). Of these, 192 (56.5%) were boys, and 148 (43.5%) were girls. This sample was split randomly into two groups to conduct the exploratory factor analysis (EFA) and confirmatory factor analysis (CFA). The randomization was carried out using a random number generator within SPSS to ensure an unbiased distribution of participants across the groups. In total, 170 participants per group were assigned, maintaining the balance between boys and girls in each group: Subsample A involved 170 students (96 boys and 74 girls), with a mean age of 17.36 years (SD = 0.78), and Subsample B involved 170 students (96 boys and 74 girls), with a mean age of 17.40 years (SD = 0.84). The groups were balanced to ensure that no confounding variables, such as age or school level, influenced the results. This approach ensures that the model’s robustness is assessed across separate subsets of data, allowing for a stronger validation of the instrument’s psychometric properties within this Italian-speaking sample.

A sample of high school students who voluntarily joined the career guidance activities at their high schools were administered an online survey that included the FTOS. The measures were delivered by career counselors with post-graduate training in career counseling and vocational guidance, following the ethical procedures established by the Italian Society for Vocational Guidance (SIO). Students were informed that their answers would be used to generate personalized reports that they would receive confidentially. Participation was voluntary, and students were free to withdraw from the study at any time. The administration phase took about 40 min.

*Ethical considerations*. All the deontological procedures provided by the Italian Society of Vocational Guidance (SIO) and the Italian Association of Psychology (2015, revised 2022) were followed. The Ethical Code of the Italian Association of Psychology draws inspiration from the Declaration of Helsinki (1964/2013).

#### 2.1.2. Procedures for Instrument Adaptation

The translation of the FTOS followed forward and backward translations of the original scale, according to the EORTC translation guidelines (Shell & Husman, 2001). Two Italian translators independently completed the forward translation and negotiated any differences between the two versions. The reconciled Italian version was then given to two English translators, who independently back-translated the measure. Any discrepancies were discussed and resolved, and modifications were made in the FTOS to consider any rewording to improve the conceptual relevance and comprehension of the items. Sample size was determined a priori to ensure an adequate fit of the FTOS, starting with a translation of the full English version, which included a two-factor model with eight manifest variables.

Then, a small focus group with 10 adolescents (5 boys and 5 girls) was convened and structured. The focus group was ‘structured’ in that it adhered to a semi-structured discussion guide, which contained questions regarding the comprehensibility, cultural appropriateness, and potential ambiguities of the FTOS items. The discussion involved guided reflections designed to encourage feedback on the clarity and relevance of each item. No literacy discrepancies or major comprehension issues arose.

#### 2.1.3. Measures

*Future Time Orientation Scale* (FTOS; Coscioni et al., 2024) is composed of 8 items, divided into two factors. The first factor, Impact, consists of five items (e.g., “I value activities that may benefit me in the long run”). The second factor, Distance, comprises three items (e.g., “Two years in the future seems to me like short period of time”). Participants were asked to indicate the extent to which they agreed with statements on a 7-point scale, from 1 (*strongly disagree*) to 5 (*strongly agree*).

*Career Adapt-Abilities Scale-Italian Form* (CAAS-Italy; Soresi et al., 2012). It consists of 24 items, the same as in the Career Adapt-Abilities Scale-International Form 2.0 (Strathman et al., 1994). Participants responded to each item on a scale from 1 (*not strong*) to 5 (*strongest*). The 24 items combine into a total score indicating career adaptability and are also divided into four subscales that measure the adaptability resources of concern (e.g., “Realizing that today’s choices shape my future”), control (e.g., “Counting on myself”), curiosity (e.g., “Investigating options before making a choice”), and confidence (e.g., “Working up to my ability”). The 24-item CAAS-Italy had good reliability ranging from 0.74 to 0.85 (see (Thomaes et al., 2023)). For this study, Cronbach’s alpha for the four subscales were 0.79, 0.70, 0.74, and 0.82, respectively. 

*Design My Future* (DMF; Di Maggio et al., 2016). It consists of 19 items on a scale from 1 (*It describes me not at all*) to 5 (*It describes me very well*). It assesses Future orientation (11 items, e.g., “Thinking about the future excites me”) and Resilience (8 items, e.g., “Even under pressure, I’m able to concentrate, to think with finish and carefully”). Previous analyses (Razavi, 2001) demonstrated construct validity and good internal consistency estimates of 0.88 for Future orientation and 0.80 for Resilience. For this study, Cronbach’s alpha for the two subscales were 0.84 and 0.85, respectively. 

#### 2.1.4. Data Analysis

In order to carry out the statistical analyses, we used the package SPSS v. 29 for the verification of the univariate and multivariate hypotheses and for the Exploratory Factor Analysis (EFA) with Maximum Likelihood (ML) and Promax rotation. In particular, we used the Kaiser criterion (eigenvalues > 1) and parallel analysis to compare the observed eigenvalues with those generated randomly. Additionally, we examined the scree plot to confirm the factor solution. SPSS was also used to assess the internal consistency through Cronbach’s raw α coefficient and McDonald’s ω coefficient. The Confirmatory Factor Analysis (CFA) used as an extraction method was performed using MPLUS. To test the adequacy of the CFA model, as suggested by the technical literature (Teo, 2009), Chi-square, CFI (Comparative Fit Index), TLI (Tucker–Lewis Index), and RMSEA (Root-Mean-Square Error of Approximation) were used as relevant fit indicators, with CFI and TLI > 0.95 and RMSEA < 0.06 as excellent model fit indicators (2002). RMSEA values below 0.08 generally indicate an acceptable model fit, while values below 0.06 indicate an excellent fit (Topoğlu, 2022). Given that our model was relatively complex and the sample size had limitations, a threshold of 0.08 was considered appropriate.

Convergent and discriminant validity were determined by comparing the correlations between measures of future time orientation, future orientation, resilience, and career adaptability, using Pearson coefficients with their Confidence Intervals (CIs). In particular, convergent validity was assessed through correlations between the subscales of the FTOS and theoretically aligned measures such as Future Orientation and Career Concern. Discriminant validity was evaluated by examining weak or non-significant correlations with constructs such as Career Control, Curiosity, Confidence, and Resilience.

### 2.2. Results

#### 2.2.1. Preliminary Analysis

Before proceeding with the Exploratory Factor Analysis (EFA), the assumptions of normality, skewness, and kurtosis were examined. Skewness values between −1 and +1 indicate acceptable normality, while kurtosis values between −2 and +2 are considered acceptable. In this analysis, all skewness and kurtosis values fell within these ranges, confirming that the data met the criteria for normality (Soresi et al., 2012).

There were no missing data in this study because the online protocol required participants to respond to all items, making it mandatory to complete the questionnaire in its entirety before submission. This ensured that all data points were collected for analysis without any omissions.

The Kaiser–Meyer–Olkin (KMO) measure of sampling adequacy was calculated to be 0.827, indicating good sampling adequacy. Additionally, Bartlett’s Test of Sphericity was conducted, yielding an approximate χ^2^(28) = 747.079, which was statistically significant (*p* < 0.001). These results support the appropriateness of conducting EFA on the dataset.

Furthermore, the correlation values between items, as shown in Table 1, revealed significant correlations ranging from moderate to strong. No issues of multicollinearity were identified, supporting the suitability of the items for factor analysis.

#### 2.2.2. Exploratory Factor Analysis (Sub-Sample A)

As regards the Exploratory Factor Analysis (EFA) model, the fit indices for the two factors indicated an overall acceptable fit to the data. The retention of factors was guided by multiple criteria, including Kaiser’s criterion (eigenvalues > 1) and a Parallel Analysis, which compared the eigenvalues of the observed data with those from randomly generated data. The Root Mean Square Error of Approximation (RMSEA) was 0.068, with a 90% confidence interval of 0.031–0.105, and the probability that RMSEA ≤ 0.05 was 0.182. This RMSEA value is within the acceptable range (<0.08), indicating an adequate fit, albeit not optimal. The Comparative Fit Index (CFI) and Tucker–Lewis Index (TLI) were both high, with values of 0.977 and 0.950, respectively. These indices are both above the 0.95 threshold, suggesting an excellent fit to the data. Additionally, the Standardized Root Mean Square Residual (SRMR) was 0.027, which is below the commonly accepted threshold of 0.05, further indicating a good model fit.

Overall, the two-factor EFA model demonstrated an adequate fit according to multiple criteria, with CFI and TLI values suggesting a strong model fit, while RMSEA and SRMR values fell within acceptable ranges. Although the Chi-Square Test of Model Fit was significant, the RMSEA, CFI, TLI, and SRMR collectively suggest that the two-factor solution adequately represents the data structure. Specifically, the first factor is composed of five items, and it is labeled Impact. The second factor is composed of three items, and it is labeled Distance. In Table 2, we report the factor loading of each item. As regards the first factor, Impact reliability for this study was Cronbach’s raw alpha (α) = 0.87; McDonald’s omega (ω) = 0.87; CIs 95% 0.84; 0.90. As regards the second factor, Distance reliability for this study was Cronbach’s raw alpha (α) = 0.73; McDonald’s omega (ω) = 0.87; CIs 95% 0.64; 0.79.

#### 2.2.3. Confirmatory Factor Analysis (Sub-Sample B)

The Root Mean Square Error of Approximation (RMSEA) was estimated at 0.08, with a 90% confidence interval ranging from 0.060 to 0.116. The probability that RMSEA is less than or equal to 0.05 was found to be 0.015, indicating a moderate level of approximation error.

Further analysis revealed a Comparative Fit Index (CFI) of 0.945 and a Tucker–Lewis Index (TLI) of 0.919, both exceeding the acceptable threshold of 0.90, which suggests a good model fit. The Chi-Square Test of Model Fit for the baseline model produced a significant result, χ^2^(28) = 637.669, *p* < 0.001. Additionally, the Standardized Root Mean Square Residual (SRMR) was calculated at 0.071, falling below the recommended cutoff of 0.08, further supporting the conclusion that the model exhibits an acceptable fit to the data.

#### 2.2.4. Convergent and Discriminant Validity (Sub-Sample B)

The convergent validity of the FTOS is supported by the significant correlations between Impact and Distance with Future Orientation and Career Concern. Impact demonstrated a moderate positive correlation with Future Orientation (*r* = 0.446, *p* < 0.01) and Career Concern (*r* = 0.266, *p* < 0.01), indicating that it effectively captures core aspects of future-oriented thinking and proactive engagement with future goals. Similarly, Distance showed a weaker but significant positive correlation with Future Orientation (*r* = 0.224, *p* < 0.05) and a weak but significant positive correlation with Career Concern (*r* = 0.162, *p* < 0.01), suggesting that it aligns with these constructs but reflects a less central or impactful dimension of future-oriented thinking compared to Impact. The discriminant validity of the FTOS is supported by the weaker or non-significant correlations of Impact and Distance with Career Control, Career Curiosity, Career Confidence, and Resilience, constructs that are related but theoretically distinct. For Career Control, Impact showed a weak but significant correlation (*r* = 0.196, *p* < 0.01), indicating some conceptual overlap, while Distance showed no significant correlation (*r* = 0.039), supporting a stronger distinction for this factor. For Career Curiosity, Impact again exhibited a weak but significant correlation (*r* = 0.181, *p* < 0.01), and Distance showed no significant association (*r* = 0.072), emphasizing clear separability. With Career Confidence, Impact demonstrated a weak significant correlation (*r* = 0.164, *p* < 0.01), whereas Distance had no significant relationship (*r* = −0.017), further underscoring its distinctiveness. For Resilience, Impact showed a moderate positive correlation (*r* = 0.343, *p* < 0.01), aligning it partially with adaptive psychological behaviors, while Distance showed no significant correlation (*r* = 0.072), reflecting its weaker association with resilience. These results confirm that both factors, especially Distance, maintain a distinct identity separate from these constructs, further supporting their discriminant validity (see Table 3).

## 3. Study 2

The second study examined the relationships between future time orientation, future orientation, and the tendency of adolescents to consider global challenges to attain sustainable development in their reflections about their future. Specifically, based on previous studies (e.g., Carmi & Arnon, 2014; Luthans et al., 2006), we hypothesized that future time orientation, intended as a general disposition toward the future, predicted directly and indirectly, through the mediational role of future time orientation in the professional field, the tendency of adolescents to consider global challenges to attain sustainable development in their reflections about their future.

### 3.1. Method

#### 3.1.1. Participants and Procedure

Participants were 170 high school students (95 boys and 75 girls), with a mean age of 17.39 years (SD = 0.79). For this study, we used the same procedure used in the first study. Specifically, the adolescents involved in this research participated in vocational guidance activities in their high schools. Before joining the vocational guidance activities, participants were informed of the goal of the research, the methods, and the expectations they could have after the end of the study. A rigorous consent procedure was followed. It involved parents’ consent and permission from school councils. Adolescents were free to not join the study.

Before the beginning of the research, the career counselors informed participants about the confidentiality of their responses and about the customized report regarding the results they would receive after the conclusion of the study.

#### 3.1.2. Measures

*Future Time Orientation Scale* (FTOS; Coscioni et al., 2024). For this study, Cronbach’s alpha for the two subscales were 0.81 and 0.75, respectively. 

*Design My Future* (DMF; Shell & Husman, 2001). In this study, only the factor Future orientation was used (Cronbach’s alpha = 0.83). 

*Goals for Future Design of the 2030 Agenda* (GFD; Santilli et al., 2023) was used to analyze the propensity to consider systemic challenges to attain sustainable development. Specifically, this scale is composed of 17 items that refer to the 17 goals presented in the 2030 Agenda for Sustainable Development. An example of an item is as follows: “In the future, there will certainly still be much to do to ensure employment and decent work for all… How could this topic of promoting decent work influence your career design?” Using a 5-point Likert scale (1 = *almost not at all*, 5 = *very much*), participants rate how much they think that every goal can affect their career. This instrument measures four factors associated with the main systemic challenges to attain sustainable development and a total score indicating the propensity to sustainability in making decisions about one’s future. The four factors measured are social/health, environment/nature, human rights and equal economic development, and policy and democracy. Santilli et al. (2023) have reported good reliability indices, with Cronbach’s alpha values of 0.70 (social/health), 0.91 (environment/nature), 0.75 (human rights and equal economic development), 0.79 (policy and democracy) for the subtests, and 0.93 for the total score. In this study, the GFD has shown adequate internal consistency reliability, with a Cronbach’s alpha of 0.92 for the total score.

#### 3.1.3. Data Analysis

A mediation analysis was conducted using the PROCESS macro for SPSS (Hayes, 2022) to examine whether future orientation mediates the relationship between future time orientation (independent variable) and the propensity to consider systemic challenges to attain sustainable development (dependent variable). Model 4 of PROCESS was specified, which is designed for simple mediation models. This model specifies that the independent variable (future time orientation) influences the mediator (future orientation), which in turn influences the dependent variable. A total of 1000 bootstrap samples were used to calculate 95% confidence intervals for the indirect effect.

### 3.2. Results

#### 3.2.1. Effect of Future Time Orientation on Future Orientation (Mediator)

The first regression model tested the effect of future time orientation on future orientation, the hypothesized mediator. This model was statistically significant, *F*(1, 84) = 18.01, *p* < 0.001, accounting for approximately 17.65% of the variance in future orientation (R^2^ = 0.18). The effect of future time orientation on future orientation was positive and significant, b = 0.78, SE = 0.18, t = 4.24, *p* < 0.001, 95% CI (0.42, 1.15), indicating that for each unit increase in future time orientation, future orientation increased by 0.78 units (see Table 4).

#### 3.2.2. Effect of Future Time Orientation and Future Orientation on Propensity to Consider Systemic Challenges to Attain Sustainable Development (Dependent Variable)

The second regression model tested the direct and indirect effects of future time orientation and future orientation on the propensity to consider systemic challenges to attain sustainable development (see Table 4). This model was also statistically significant, *F*(2, 83) = 11.24, *p* < 0.001, explaining 21.32% of the variance in the propensity to consider systemic challenges (R^2^ = 0.21). The effect of future orientation on the propensity to consider systemic challenges was positive and statistically significant, b = 0.51, SE = 0.12, t = 4.13, *p* < 0.001, 95% CI (0.26, 0.75), suggesting that each unit increase in future orientation was associated with a 0.51 unit increase in the propensity to consider systemic challenges. However, the direct effect of future time orientation on the propensity to consider systemic challenges was not statistically significant, b = 0.09, SE = 0.23, t = 0.39, *p* = 0.700, 95% CI (−0.37, 0.54).

#### 3.2.3. Indirect Effect of Future Time Orientation on Propensity to Consider Systemic Challenges to Attain Sustainable Development Through Future Orientation

A bootstrap analysis (1000 samples) indicated that the indirect effect of future time orientation on the propensity to consider systemic challenges to attain sustainable development through future orientation was significant, b = 0.40, BootSE = 0.13, 95% CI (0.17, 0.68). Since the confidence interval does not include zero, the indirect effect is statistically significant, suggesting that future orientation significantly mediates the relationship between future time orientation and the propensity to consider systemic challenges to attain sustainable development.

## 4. General Discussion

The goal of this article was to describe the adaptation and validation of the FTOS in the Italian context as a measure of future time orientation. For this purpose, we conducted two studies.

In the first study, we provided evidence of validity and reliability for the FTOS by examining its internal structure with exploratory factor analysis and confirmatory factor analysis, with two independent sub-samples. More specifically, in line with the original version of the instrument (Kurth & Pihkala, 2022), two factors were detected. The first one, named Impact, included five items concerning the inclination to be influenced by one’s psychological future during current decisions and behaviors. The second factor, named Distance, involved three items concerning the perception of time in the future.

The analysis of the correlations between the two factors of the Future Time Orientation Scale (FTOS), Impact and Distance, alongside their relationships with Resilience and the Career Adaptability Inventory (CAI), provides valuable insights into the scale’s convergent and discriminant validity. The convergent validity is reflected in the meaningful relationships between the FTOS factors and theoretically aligned constructs, such as Future Orientation and Career Concern. The results support the notion that future-oriented thinking involves both a cognitive temporal perspective and proactive engagement with future goals, as outlined in theories of future orientation. The discriminant validity of the FTOS is evidenced by its weaker or non-significant correlations with constructs such as Career Control, Career Curiosity, Career Confidence, and Resilience, which are related but conceptually distinct. This aligns with theoretical distinctions between future orientation and constructs tied to career adaptability and psychological resilience (Savickas & Porfeli, 2012). While future time orientation involves goal-directed planning and anticipation, career adaptability dimensions such as Career Control and Career Curiosity emphasize situational flexibility and exploration, reflecting different psychological processes. The stronger correlation between Impact and Resilience, compared to Career Concern, may be due to the broader applicability of resilience across various life domains, whereas Career Concern is more narrowly focused on specific aspects of professional development that might be less salient for adolescents. Additionally, since previous research identified these associations primarily in adult populations, developmental differences could explain the weaker relationship observed in adolescents. Resilience functions as a general measure of psychological adaptation, intersecting only partially with future orientation, which is more goal-directed and anticipatory in nature (Coscioni et al., 2024; Zimbardo & Boyd, 1999).

Theoretical models posit that future-oriented behaviors are driven by an individual’s ability to anticipate and prepare for future challenges, and these findings seem to reinforce the central role of these constructs within the FTOS framework (Zimbardo & Boyd, 1999; Hayes, 2022). The relationships observed underscore the capacity of the FTOS to capture core aspects of future-oriented cognition and motivation.

Overall, these findings provide preliminary support for the validity of the FTOS, particularly the Impact factor, which shows robust associations with well-established constructs such as Resilience and Career Adaptability. However, the results also suggest that further refinement of the Distance factor may be necessary to enhance the scale’s ability to capture the complex dimensions of future time orientation. Future research should continue to explore the relationship between these factors and other psychological constructs to better understand the underlying mechanisms of future-oriented thinking and its impact on personal and career development.

With the second study, we wanted to evaluate a partial mediational model between future time orientation and the tendency of adolescents to consider global challenges to attain sustainable development in their reflections about their future through future orientation in the professional field. The analyses partially confirmed the hypotheses. More specifically, future orientation fully mediated the relationship between future time orientation and the tendency of adolescents to consider global challenges to attain sustainable development in their reflections about their future. This result is in line with the previous literature that underlined the role of future orientation both for the assumption of sustainable behaviors (e.g., Carmi & Arnon, 2014) and for the intentions and career choices for the benefit of sustainability (e.g., Thelken & de Jong, 2020). A possible explanation for this relationship is connected to the fact that higher levels of future orientation lead to higher concerns regarding one’s individual and collective future compared to more present-anchored mindsets. More future-oriented individuals are more able to analyze globally relevant matters at a higher level of abstraction. They are more aware and, thus, could be more able to foresee potential future negative consequences if social and environmental global challenges are not addressed (Valenzuela-Fernández et al., 2022; Santilli et al., 2023). Accordingly, future-oriented adolescents are more inclined to sustainability when making career decisions about their future.

This research has also underlined that future time orientation was significantly related to future orientation, which, in turn, was positively related to the tendency of adolescents to consider global challenges to attain sustainable development in their reflections about their future. In contrast with the hypotheses, no direct relationship between future time orientation and the tendency of adolescents to consider global challenges to attain sustainable development in their reflections about their future has been detected. A possible explanation could be that the general disposition toward the future (future time orientation) may positively impact the ideas, thoughts, and feelings of adolescents regarding their professional future (future orientation), thus promoting their tendency to consider environmentally and socially sustainable career choices. Previous studies examined the impact of future time orientation as a trait or general disposition on sustainable behaviors in an extensive way in everyday life and its different areas (Carmi & Arnon, 2014); in this study, we instead considered a specific outcome related to sustainability, that is, the propensity to sustainability in making a career decision about one’s future. It may be considered a specific field in which future orientation, as a manifestation of future time orientation on specific tasks, seems to act directly (Coscioni et al., 2024; Di Maggio et al., 2016).

## 5. Theoretical and Practical Implications

This paper has both theoretical and practical implications. From a theoretical perspective, the FTOS represents a valid and reliable tool that can support researchers and practitioners in the analysis of the psychological future during adolescence through a multidimensional approach that consists of a more complete analysis of the psychological future compared to other instruments with a single factor focused on the future (Coscioni et al., 2024).

Moreover, this study underlined, for the first time, the role of future time orientation on future orientation in the professional context. This, in turn, impacts the propensity to sustainability when making career decisions about the future, suggesting the importance of future orientation not only for the assumption of daily behaviors that support sustainability but also for adolescents’ reflections regarding their educational and professional future in terms of career choices to address global, social, and environmental challenges (Commodari & La Rosa, 2020; Carmi & Arnon, 2014).

From a practical perspective, the FTOS can be used during individual career counseling activities and career interventions to evaluate adolescents’ psychological futures. Specifically, it can be useful to stimulate reflections with adolescents about their inclination to be affected by the psychological future in current decisions and behaviors and their perception of time in the future. The FTOS can enable intraindividual and inter-individual assessments of future time orientation in adolescents and can be administered at pre- and post-tests to evaluate the efficacy of career interventions aimed at promoting adolescents’ future time orientation.

Moreover, the mediational model identified showed that the propensity to sustainability in making career decisions about the future can be influenced by future orientation and future time orientation. This means that it is necessary to design career guidance activities that promote future orientation and future time orientation to make adolescents reflect on the current context and some problems and threats to the planet connected to social and environmental risks and encourage them to seriously consider the planet’s future and deepen their comprehension of the relationship between current career choices and future development. This may represent fertile ground to encourage young people to make career choices for the benefit of sustainability (Carmi & Arnon, 2014; Rochat, 2024).

## 6. Limitations and Future Directions

The present paper has some limitations. Firstly, the predictive validity and the test–retest reliability of the FTOS have not been tested. Future studies may consider the possibility of conducting these analyses. Secondly, we do not have information regarding the socio-economic status and family background of participants, which could impact their future time orientation. This information should be considered in future studies. Thirdly, we did not control demographic variables (e.g., age and sex) in the mediational model for the second study. Future studies should consider a more comprehensive analysis of these potential demographic influences (e.g., age, gender) to provide a fuller understanding of the factors impacting adolescents’ future time orientation and sustainable career choices. Fourthly, another limitation is related to potential bias due to the self-report nature of the instruments and the cross-sectional design (e.g., common method variance, social desirability bias). Future studies could address these issues using longitudinal designs, as these significantly enhance the interpretation of causality (Razavi, 2001).

## 7. Conclusions

This paper describes the adaptation and validation of the FTOS to the Italian context, ensuring its effectiveness in measuring psychological future orientation among Italian-speaking adolescents. The two studies conducted on samples of Italian adolescents showed that the FTOS is a valid and reliable instrument that can be used during counseling and research activities to assess future time orientation in adolescents.

## Figures and Tables

**Table 1:** Item correlations.

Item	1	2	3	4	5	6	7	8
1	-	0.335 **	0.115	0.569 **	0.358 *	0.242 **	0.289 **	0.249 **
2		-	0.551 **	0.402 **	0.065	0.667 **	0.598 **	0.614 **
3			-	0.303 **	0.156 *	0.484 **	0.347 **	0.398 **
4				-	0.358 *	0.396 **	0.372 **	0.369 **
5					-	0.170 *	0.182 **	0.198 **
6						-	0.731 **	0.733 **
7							-	0.712 **
8								-

** The correlation is significant at the 0.01 level (two-tailed). * The correlation is significant at the 0.05 level (two-tailed).

**Table 2:** Factor loading matrix of the Italian version of FTOS.

Item	Factor 1	Factor 2	Communality (h^2^)
Item 6	0.887	0.155	0.787
Item 8	0.830	0.151	0.689
Item 7	0.798	0.210	0.637
Item 2	0.749	0.013	0.561
Item 3	0.544	0.120	0.296
Item 4	0.147	0.672	0.452
Item 1	0.127	0.651	0.424
Item 5	0.036	0.374	0.140

**Table 3:** Correlations between future orientation, resilience, impact, distance, and the career adaptability inventory.

	Impact	Distance	Career Concern	Career Control	Career Curiosity	Career Confidence	Future Orientation	Resilience
**Impact**	-	0.196 **	0.266 **	0.196 **	0.181 **	0.164 **	0.446 **	0.343 **
**Distance**	0.417 **	0.039	0.162 **	0.039	0.072	−0.017	0.224 *	0.072

Note: *p* < 0.01 (**), *p* < 0.05 (*).

**Table 4:** Results of mediation analysis using PROCESS Model 4.

Predictor	b	SE	t	*p*	95% CI
**Direct Effect** **(FTOS → GFD)**	0.0885	0.0807	1.10	0.6998	−0.0706, 0.2476
**Mediator** **(Time P. → GFD)**	0.5079	0.1262	4.02	<0.001	0.2589, 0.7569
**Indirect Effect** **(FTOS → GFD, mediated by Time P.)**	0.3967	0.1334	-	<0.001	0.1666, 0.6790
**Intercept**	364.179	21.767	16.74	<0.001	32.0883, 40.7475

## Data Availability

The data used in this research are unavailable due to privacy and ethical restrictions.
